# The Origin and Molecular Epidemiology of Dengue Fever in Hainan Province, China, 2019

**DOI:** 10.3389/fmicb.2021.657966

**Published:** 2021-03-24

**Authors:** Lin Liu, Tao Wu, Biao Liu, Rajaofera Mamy Jayne Nelly, Yumei Fu, Xun Kang, Chuizhe Chen, Zenyan Huang, Biao Wu, Jiao Wang, Zhongyi Zhu, Jinmin Ma, Ming Liu, Yanru Zhang, Chuanyu Bao, Feng Lin, Weijun Chen, Qianfeng Xia

**Affiliations:** ^1^NHC Key Laboratory of Control of Tropical Diseases, Key Laboratory of Tropical Translational Medicine of Ministry of Education, School of Tropical Medicine and Laboratory Medicine, Hainan Medical University, Haikou, China; ^2^Department of Infectious Diseases, Hainan General Hospital, Hainan Affiliated Hospital of Hainan Medical University, Haikou, China; ^3^BGI PathoGenesis Pharmaceutical Technology, BGI-Shenzhen, Shenzhen, China; ^4^BGI Education Center, University of Chinese Academy of Sciences, Shenzhen, China; ^5^Department of Emergency Surgery, Hainan General Hospital, Hainan Affiliated Hospital of Hainan Medical University, Haikou, China

**Keywords:** dengue fever, molecular-characteristics, bayesian phylogenies, hainan province, epidemiological

## Abstract

There was an outbreak of Dengue fever on September 5, 2019, in Hainan Province, which has not been endemic for 28 years. We aim to describe the clinical and epidemiological features of the 2019 outbreak in Hainan Province and identify the cause. All type 1 Dengue fever cases that occurred in this outbreak of Hainan exhibited mild clinical symptoms. The epidemiological investigations indicate that the outbreak might originate from workers in the Xiuying area, Haikou City, form a concentrated outbreak, and then spread out. Bayesian phylogenies results and epidemiological data were used to infer a likely series of events for the dengue virus’s potential spread and trace the possible sources. The strains’ sequences were close to a sequence from the nearby Guangdong province, supporting the hypothesis that the dengue virus was imported from Guangdong province and then spread across Hainan province. Furthermore, it is interesting that two other strains didn’t group with this cluster, suggesting that additional introduction pathways might exist. The study indicated that the dengue fever epidemic presented two important modes in Hainan. Firstly, epidemics prevalence was caused by imported cases, and then endogenous epidemics broke out in the natural epidemic focus.

## Introduction

Dengue fever (DF), a mosquito-borne viral infection common in warm, tropical climates, is an acute infectious disease caused by the Dengue virus (DENV). Since the first reported outbreak in 1779 in Jakarta, Indonesia, outbreaks have frequently been found in tropical and sub-tropical climates worldwide, mostly in urban and semi-urban areas with local variations in risk influenced by rainfall, temperature, and relative humidity (Guzman et al., 2013; Halstead and Cohen, 2015; Niyati and Ira, 2016). The incidence of dengue has grown dramatically around the world in recent decades. One study on dengue prevalence estimates that 3.9 billion people are at risk of dengue viruses (Brady et al., 2012; Bhatt et al., 2013). Despite a risk of infection in 128 countries, 70% of the actual burden is in Asia.

The first recorded outbreak of DF in China occurred in Guangdong province in 1978. It was caused by DENV-3 imported from countries in south-east Asia. Since then, imported dengue fever cases have often caused local outbreaks in provinces like Guangxi, Yunnan, Hainan, Fujian, etc., in China (Wang et al., 2018; Zhang et al., 2019). Two large outbreaks of dengue virus type 1 and dengue virus type 3 were recorded, respectively, during 2006–2007 and 2012–2015 (Sang et al., 2014; Zhang et al., 2019). Hainan is the southernmost province and the second largest island in China, with Guangdong province across the Qiongzhou Strait to the north. It is a tropical monsoon climate with a rainy season from May to October. The meteorological conditions are favorable for the breeding of *Aedes* mosquito larvae and the transmission of DF. There were three DF epidemics in the epidemiological history of Hainan. The first one was caused by dengue virus type 3 in 1978 (Fan et al., 1989). Then there was a second struck in 1985–1988, and a third one, a dengue hemorrhagic fever in 1991, both caused by dengue virus type 2 (Qiu et al., 1991). Ever since 1991, there were no more outbreaks.

In August 2019, several workers in the Wuyuan River area of Xiuying District, Haikou City, experienced fever, fatigue, and rashes. After further testing for NS1 antigen and dengue virus nucleic acid, the first case of dengue fever was diagnosed in Hainan General Hospital on September 5, 2019. Subsequently, close contacts of confirmed cases and those with suspicious exposures to the Wuyuan River area were screened, and a follow-up study by health professionals found multiple suspicious fever cases around the epidemic site. The Hainan Centre for Disease Control conducted epidemiological investigations, sampling inspections, and mosquito control treatments on the epidemic site. After further testing for NS1 antigen and dengue virus nucleic acid, multiple DF cases were diagnosed, and the local outbreak of dengue fever was confirmed. Altogether 5 cities and towns throughout the province reported the local epidemic situation (Haikou, Wenchang, Danzhou, Lingshui, and Wanning). A total of 264 confirmed dengue cases were reported in Hainan Province from September 5, 2019 to October 17, 2019.

In this study, we described the clinical and epidemiological features of the 2019 dengue fever outbreak and determined the complete genome sequences of 6 dengue isolates collected and E gene sequences from 90 patients in Hainan during the epidemic. In combination with those published sequences in GenBank, we conducted an extensive molecular epidemiological analysis to determine where the DENV isolates in Hainan originally came from and what shaped their evolution, which is important to dengue control and prevention.

## Materials and Methods

### Collection of Isolates and Clinical Information

According to the Chinese Center for Disease Control and Prevention in 2018 issued by the Dengue Fever Prevention and Control of Technical Guidelines and Criteria for the diagnosis of dengue (WS216 2018), an outbreak of dengue fever was definition in Hainan. The dengue diagnosis was confirmed based on the epidemiological history, clinical manifestations, and laboratory examination. The serum samples from confirmed cases at the different infection times and different areas (from Haikou, Danzhou, Wanning, Lingshui, Wencang cities) were collected for dengue virus gene sequencing and E gene analysis. In this epidemiological report, we used data from September 5, 2019 to October 17, 2019, to describe the disease’s clinical and epidemiological features and explore where the DENV virus in Hainan originally came from and what shaped their evolution.

### Laboratory Diagnostic Assays

A total of 6 serum samples were selected for whole-genome sequencing, and 90 DENV-positive sera were collected to amplify and sequence the E gene by The Beijing Genomics Institute (BGI). Sequence-dependent single primer amplification(SDSPA)was used to enrich the viral nucleic acid, construct the sequencing library, and PE150 strategy was selected for sequencing on the MGI-2000 sequencing instrument (BGI, CHN). Nucleic acids were extracted using QIAamp Viral RNA Mini Kit (52904#, QIAGEN).

### Phylogenetic Analysis

Multiple sequence alignment was done by MUSCLE (V3.8.31) with a maximum number of iterations of 16. The maximum likelihood (ML) phylogenetic trees were estimated by FastTree (Version: 2.1) of multiple alignment sequences, with nucleotide distances calculated using the Jukes-Cantor nucleotide substitution model and branch lengths inferred by CAT approximation with 20 rate categories. Final ML trees (both the topology and the branch lengths) were improved by Normal + NNI + SPR (2 rounds range 10) + ML-NNI opt-each heuristic tree search algorithms. Local support values with 1,000 replicates calculated by the Shimodaira-Hasegawa test (SH-like local supports) were estimated to assess each split’s robustness in the tree.

### Molecular Dating Using BEAST

Bayesian phylogenies were performed in BEASTv.2.6.0 (Bouckaert et al., 2019). The substitution model was estimated by the J model test (V2.1.10) to access the most suitable nucleotide model with the default parameter to access 88 potential-based substitution models. The GTR + G model is selected based on best fit Bayesian information criterion (BIC) score evaluated by J model test. A strike clock rate combined with Coalescent Constant Population model was used to evaluate the most recent common ancestor (MRCA) prior to each clade (Russel et al., 2019). Finally, Tree Annotator v2.5.2 was applied to summarize Maximum-clade credibility trees of all MCMC trees with posterior probability values reported in each node.

## Results

### Epidemiology

**FIGURE 1:**
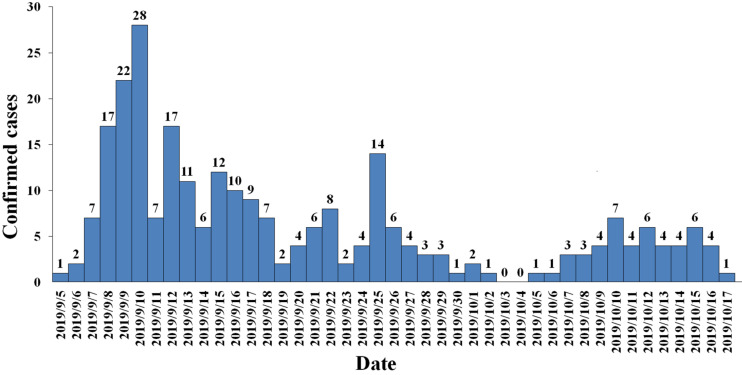
Daily trend chart of confirmed dengue fever cases.

**FIGURE 2:**
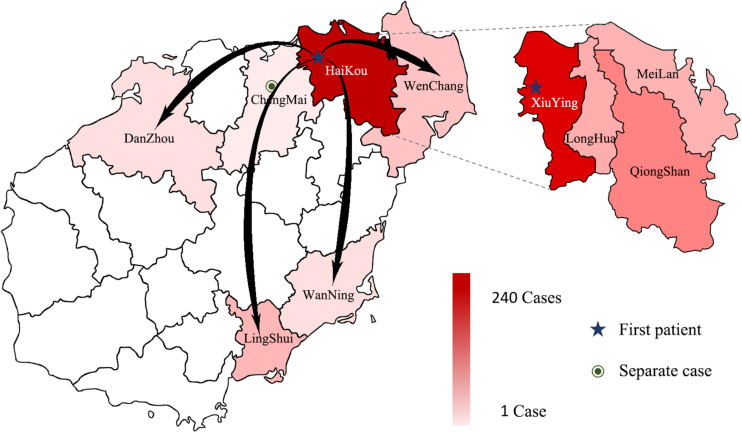
The distribution of DENV in Hainan in 2019. The intensity of the colors in the figure indicates the number of dengue cases; the darker the color is, the more dengue cases there were in the area, arrows indicate the possible direction of propagation, dotted lines show the four Districts of Haikou city.

Between September 5, 2019 and October 17, 2019, 3941 suspected cases were identified, 264 cases were confirmed dengue fever with PCR positive and NS1 antigen positive in hospital. From September 5 to September 13, a total of 105 cases were reported, including 95 cases in Wuyuan River area of Xiuying District and the number of confirmed cases of dengue fever peaked on September 10 (Figure 1). Infection primarily occurred in the Xiuying District in Haikou city (182 in Xiuying district, 36 in Qiongshan district, 13 in Longhua district, and 9 in Meilan district), and the remaining cases were distributed in several other cities in Hainan province, 7 in Wenchang city, 3 in Danzhou city, 10 in Lingshui city, 3 in Wanning city, and 1 in Chengmai city (Figure 2).

### Clinical Features

In this epidemic, we retrospectively analyzed the clinical characteristics and laboratory findings of patients diagnosed with dengue fever. On the first day of admission, most of the patients had a fever (97.1%), headache (81.5%), and muscle ache (56%), and their body temperature was usually between 38 and 39°C, which could be reduced by themselves. As the course of the disease prolongs, fever, headache, muscle aches, and other symptoms can gradually disappear, and a few patients have abdominal pain (1.4%), diarrhea (7%), and rash (2.8%), but the symptoms are mild, without ecchymosis, bleeding, and hepatomegaly (Table 1).

**TABLE 1:** List of clinical manifestations of 141 patients with DF.

Symptom days	Fever	Headache	Rash	Muscle ache	Abdominal pain	Diarrhea
Day 1	137 (97.1%)	115 (81.5%)	4 (2.8%)	79 (56%)	2 (1.4%)	10 (7.0%)
Day 2	93 (65.9%)	83 (58.8%)	7 (4.9%)	57 (40%)	2 (1.4%)	4 (2.8%)
Day 3	78 (55.3%)	68 (48.2%)	4 (2.8%)	44 (31.4%)	1 (0.7%)	3 (2.1%)
Day 4	67 (47.5%)	57 (40%)	0	37 (26.2%)	1 (0.7%)	2 (1.4%)
Day 5	44 (31.2%)	34 (24.1%)	1 (0.7%)	23 (16.3%)	0	1 (0.7%)
Day 6	33 (23.4%)	26 (18.4%)	2 (1.4%)	11 (7.8%)	0	1 (0.7%)
Day 7	16 (11.3%)	13 (9.0%)	3 (2.1%)	4 (2.8%)	0	3 (2.1%)

62 patients showed a decrease in White Blood Cells (WBC). The WBC valley appears on the 2nd to 5th day of the disease course. As the patient’s body temperature is normal, the symptoms disappeared, and the WBC returned to normal. Eighteen patients showed mild transaminase elevation at the beginning of the admission. With normal body temperature and liver protection treatment with reduced glutathione and other drugs, the transaminase returned to normal when discharged. During the hospitalization, all patients’ albumin decreased to varying degrees but remained above 35 g/L. Fifty-five patients had a slight increase in CK and CK-MB upon admission. With normal body temperature, the patient’s clinical muscle soreness symptoms were improved, and CK and CK-MB levels gradually dropped and returned to normal when discharged (Table 2). There was neither severe dengue nor death among the DENV-infected individuals.

**TABLE 2:** Laboratory findings in patients with DF.

Laboratory findings	The first day of admission	Discharged
**Complete blood count**		
WBC(10^9/L)	4.2 ± 1.93	4.1 ± 1.88
PLT(10^9/L)	139.8 ± 74.4	229.7 ± 56.9
HCT (Male)	0.427 ± 0.39	0.434 ± 0.09
HCT (Female)	0.393 ± 0.04	0.381 ± 0.03
**Liver and kidney function**		
ALT(U/L)	31.2 ± 23.3	33.3 ± 14.9
AST(U/L)	46.5 ± 45.2	29.5 ± 28.8
TBil(umol/L)	17.8 ± 10.9	10.4 ± 7.4
ALB(g/L)	47.6 ± 9.8	41.5 ± 11.3
Cr(umol/L)	63.7 ± 30.1	81.1 ± 33.4
**Creatine kinase and C-reactive protein**		
CK(U/L)	278.8 ± 162.9	146.7 ± 122.5
CK-MB(U/L)	53.6 ± 44.6	38.7 ± 28.8
CRP(mg/L)	28.7 ± 25.6	26.8 ± 21.1

*WBC, white blood cells; PLT, platelets; HCT, hematocrit; ALT, glutamic pyruvic transaminase; AST, glutamic oxalacetic transaminase; TBil, total bilirubin; ALB, albumin; Cr, creatinine; CK, creatine kinase; CRP, c-reactive protein; CK-MB, creatine induced enzymes.*

### Phylogenetic Tree of DF-1 by Whole-Genome Sequences

The datasets presented in this study can be found in online repositories. The names of the repositories and accession numbers can be found in Supplementary Table S1.

**FIGURE 3:**
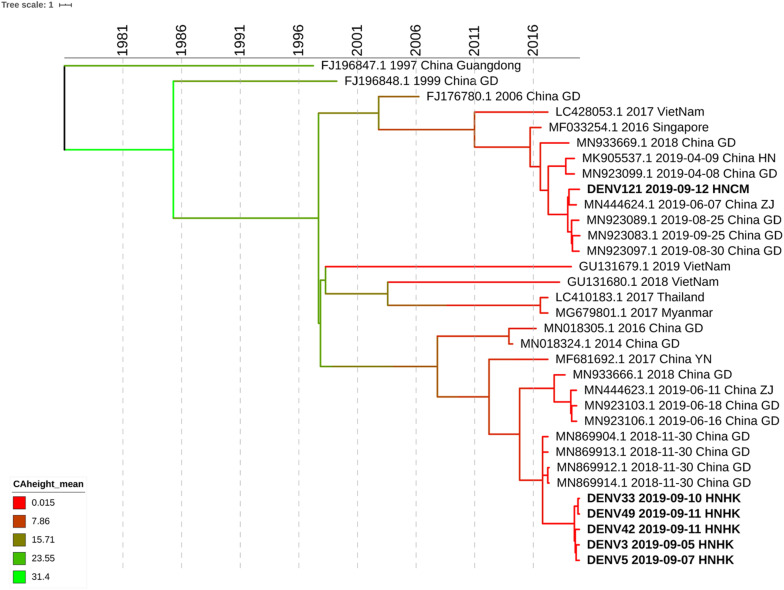
Phylogenetic tree of DF-1 by whole-genome sequences. Bayesian phylogenies were performed in BEASTv.2.6.0. Used Bayesian Skyline plus stick model setting to run Beast and identified the maximum clade credibility (MCC) tree. Analyses were run in duplicate for 100 million MCMC steps, sampling parameters, and trees were logged every 5,000 steps.

We select 5 subjects at the different infection times and different streets of dengue fever outbreak from Haikou city and 1 subject from Chenmai city who had no contact with other subjects. Whole-genome sequencing was attempted on six samples, and sequences were successfully generated. To get an accurate genome-wide tree structure, we first downloaded Dengue Type 1 genome-wide sequences at NCBI and tagged the data source and time. The earliest whole genome sequence of Asian Dengue Type 1 can be traced back to China’s Guangdong sequence in 1991 (FJ196845.1). We constructed a genome-wide BEAST phylogenetic tree from 1991 to 2020 (Figure 3).

The evolutionary analysis showed that the evolutionary tree was divided into two clades, and the homology of the six dengue virus isolates was more than 99% except for DENV121. Whole-genome sequence evolutionary analysis showed that the homology of the 5 virus strains was 99.67% as high as that of Guangzhou’s virus strains in November 2018, such as MN869904.1. DENV3, DENV5, DENV33 DENV42, and DENV49 were from the Haikou city, and case DENV121 was from Chengmai city, which was a different area from Haikou city. According to the evolutionary tree analysis, Hainan’s cases were more closely related to the dengue virus outbreak in Guangzhou and Zhejiang in June 2019. It is possible that the dengue virus has been imported from Guangzhou, Zhejiang, and other places, hiding in the crowd, and entered the dengue outbreak period at the end of August, forming a local outbreak in Hainan. According to the result of whole-genome sequencing, the homology of DENV121 and the other 5 hospitalized strains is only 96.64%, which is in two small branches, and no hospitalized link has been found between this case and other site cases. Although DENV121 has high homology with the Zhejiang strain on June 7, 2019 (MN444624.1), investigations indicate that the patient returned to Hainan from Guangzhou Province Shenzhen city in early September and the 99.94% homology with the Guangdong province’s epidemic strain on August 30, 2019 (MN923097.1). Based on homology and patient travel history, DENV121 cases were confirmed as imported isolated cases from Guangdong Province.

### Phylogenetic Tree of DF-1 by E Gene Sequences

To investigate the molecular epidemiology of dengue viruses in Hainan, a total of 90 dengue cases were collected, and E gene sequences were obtained.

**FIGURE 4:**
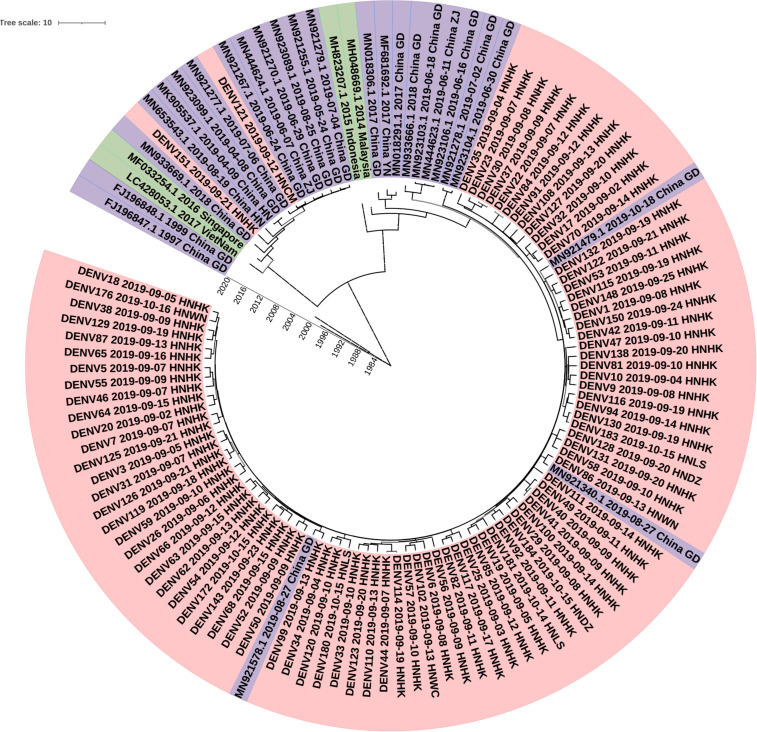
Phylogenetic tree of DF-1 by E gene sequences. China GD, Guangdong Province in China; China YN, Yunnan Province in China; China ZJ, Zhejiang Province in China; China HN, Henan Province in China; HNHK, Haikou city Hainan Province in China; HNCM, Chengmai city Hainan Province in China; HNDZ, Danzhou city Hainan Province in China; HNLS, Lingshui city Hainan Province in China; HNWN, Wanning city Hainan Province in China.

To determine the DENV molecular epidemiology in Hainan, the DNA sequences of E genes were downloaded from the NCBI compared with our 90 E gene sequences. 147 E gene sequences were used to infer BEAST phylogenetic trees (Figure 4), including 90 E gene sequences determined in the study (Supplementary Tables S1, S2) and 29 representative sequences from GenBank referring to diverse time and geographic localities. The sequence of the E gene is scattered due to a large number of samples. Eighty-eight of the Ninety clades were clustered into one clade and the other clade. The dengue viruses detected in Hainan Province showed that Guangzhou strains were likely introduced to Haikou city first and then transmitted into its neighbor cities: Linshui, Wenchang, Danzhou, and Wanning. The DENV121 and DENV151 were not closely related to other Hainan strains. Although DENV151 is closely homologous to the Henan strain, the more likely transmission route is consistent with DENV121, from Singapore to Guangdong to Hainan.

## Discussion

The World Health Organization (WHO) has recently listed dengue as a potential threat among ten diseases for 2019, and outbreaks in many countries confirm this observation (Alves, 2019). Recent more than 2.7 million cases and 1,206 deaths from January to October 2019, of which over 1.2 million were laboratory-confirmed, and more than 22,000 were classified as severe dengue by the Pan American Health Organization. Despite the increase in case numbers, the proportion of deaths in dengue cases was 26% less in 2019.

There are three DF outbreaks in Hainan province in history. In the past few decades in China, although imported cases of dengue fever often caused local outbreaks in some provinces (Wang et al., 2018; Zhang et al., 2020), no massive outbreak was reported from the island province of Hainan since 1991 (Wu et al., 2010; Jing et al., 2012; Luo et al., 2012; Shen et al., 2015). On September 5, 2019, an outbreak of dengue fever occurred in Hainan Province, which has not been endemic for 28 years. We aim to describe the clinical and epidemiological features of the 2019 outbreak in Hainan Province and identify the cause.

The dynamics of dengue transmission depend on hosts, viruses, vectors, and environmental factors. Given the special geographical conditions in Hainan and the restricted range of mosquito flying distance (Harrington et al., 2005; Maciel-De-Freitas et al., 2010), the increase in tourist and migrant workers may play a critical role in the spread of dengue. At broad spatial scales, human movements may make dengue virus being introduced and reintroduced into a region with lower herd immunity (Stoddard et al., 2009). Based on the patients’ epidemiological investigations and clinical manifestations, we initially determined that this outbreak was a local dengue fever outbreak caused by imported cases. We speculated that this outbreak originated from workers in Wuyuan River, Xiuying District, Haikou city, an urban area with a high population density and mobility, spread to the neighboring districts, caused a concentrated outbreak, and then spread to other cities.

Although our colleagues have reported the E sequences of the nine isolated strains were closely related to the Zhejiang strain (2017, MH010601), due to the lack of clinical cases and sequencing samples, the origin and molecular epidemiological features of the dengue outbreak in Hainan Province in 2019 could not be fully revealed (Du et al., 2021). We performed the phylogenetic analysis of genome sequences of dengue viruses collected during the 2019 epidemic in Hainan. It was observed that 88 sequenced strains were close to a sequence from the nearby Guangdong province, supporting the hypothesis that the dengue virus was imported from Guangdong province and then spread across Hainan province. Furthermore, it is interesting that two other strains didn’t group with this cluster, suggesting that additional introduction pathways might exist. The study indicated that the dengue fever epidemic presented two important modes in Hainan. Firstly, the endemic prevalence was caused by imported cases, and then endogenous epidemics broke out in the natural epidemic focus. Molecular epidemiological analysis of the dengue virus is important for understanding the dynamic of circulation and for risk assessment.

There are also several possible factors contributing to this outbreak in Hainan, including high mosquito population levels, favorable air temperatures, precipitation, and humidity in the summer-autumn season, all of which are suitable for the survival and breeding of mosquito vectors, as well as the incubation of the dengue virus (Degnan and Salter, 2005; Maciel-De-Freitas et al., 2010; Sang et al., 2014). From August 1 to September 15, 2019, Hainan’s average temperature was 25.4 to 29.1°C, and the average relative humidity was 77–88%. The proximity of mosquito vector breeding sites to human habitation is a significant risk factor for dengue and other diseases that *Aedes* mosquitoes transmit. These factors could provide environments that are even more conducive for DENV transmission and, hence, more frequent and larger epidemics can be expected in the coming years (Ooi, 2015).

The outbreak lasted from September to October, and the Hainan Provincial government launched an emergency response against the outbreak. Hainan’s main dengue surveillance procedure was implementing timely, appropriate laboratory confirmation, clinical management to combat the mosquito vectors (Zhang et al., 2018). As a result, the epidemic was generally under control, and few people were infected. The rapid spread and continued outbreaks of dengue show that sustained prevention and control measures should be supported by robust surveillance. Communities play a major role in the success and sustainability of vector control.

In this study, we conducted the molecular epidemiologic and phylogenetic analysis and reported interesting findings of the introduction and spread of dengue in a specific island context. The results provided an overview of the genetic diversity of DENV stains in Hainan, which also gave information about the geographic distribution, dynamic transmission, and molecular evolution of DENV stains in epidemic regions. In addition, the continuous surveillance on the imported dengue case could benefit the prevention of further dengue outbreaks in Hainan.

## Data Availability Statement

The datasets presented in this study can be found in online repositories. The names of the repository/repositories and accession number(s) can be found in the article/Supplementary Material.

## Ethics Statement

The studies involving human participants were reviewed and approved by the Review Board of Hainan General Hospital. The patients/participants provided their written informed consent to participate in this study.

## Author Contributions

FL, WC, and QX conceived and designed the experiments. LL and TW performed the experiments and analyzed the data. BL, RN, YF, XK, CC, ZH, BW, JW, ZZ, JM, ML, YZ, and CB contributed reagents, materials and analysis tools. LL wrote the manuscript. All authors contributed to the article and approved the submitted version.

## Conflict of Interest

ZZ, JM and WC are employed by company BGI PathoGenesis Pharmaceutical Technology. The remaining authors declare that the research was conducted in the absence of any commercial or financial relationships that could be construed as a potential conflict of interest.

## Supplementary Material

Supplementary Table 1 Information of 90 dengue viruses in our study.

Supplementary Table 2 The E gene primers and probes.
