# Supplementary material for: The Origin and Molecular Epidemiology of Dengue Fever in Hainan Province, China, 2019
Source: Front Microbiol. 2021 Mar 24;12:657966. doi: 10.3389/fmicb.2021.657966 (PMC8025777; doi:10.3389/fmicb.2021.657966)
Supplement: Supplementary Table 1 — Information of 90 dengue viruses in our study. [file Table_1.docx]

**Supplementary table S1**

| **Information of the E gene sequence** | | | | | | |
| --- | --- | --- | --- | --- | --- | --- |
| **Number** | **Gender** | **Address** | **DENV type** | **Collect time** | **Submissions** | **GenBank** |
| DENV1 | male | Hainan province, Haikou city, Xiuying area | 1 | 08-Sep-19 | SUB8973919 | MW541836 |
| DENV6 | female | Hainan province, Haikou city, Xiuying area | 1 | 08-Sep-19 | SUB8990053 | MW547410 |
| DENV7 | male | Hainan province, Haikou city, Xiuying area | 1 | 07-Sep-19 | SUB8990183 | MW548583 |
| DENV9 | male | Hainan province, Haikou city, Xiuying area | 1 | 08-Sep-19 | SUB8990367 | MW547421 |
| DENV10 | male | Hainan province, Haikou city, Xiuying area | 1 | 04-Sep-19 | SUB8990494 | MW548588 |
| DENV17 | male | Hainan province, Haikou city, Xiuying area | 1 | 02-Sep-19 | SUB8990593 | MW547422 |
| DENV18 | male | Hainan province, Haikou city, Xiuying area | 1 | 05-Sep-19 | SUB8990667 | MW548591 |
| DENV19 | male | Hainan province, Haikou city, Xiuying area | 1 | 05-Sep-19 | SUB8990742 | MW547423 |
| DENV20 | female | Hainan province, Haikou city, Xiuying area | 1 | 02-Sep-19 | SUB8990822 | MW547424 |
| DENV22 | male | Hainan province, Haikou city, Xiuying area | 1 | 07-Sep-19 | SUB8990886 | MW547425 |
| DENV23 | male | Hainan province, Haikou city, Xiuying area | 1 | 07-Sep-19 | SUB8991001 | MW548590 |
| DENV25 | male | Hainan province, Haikou city, Xiuying area | 1 | 03-Sep-19 | SUB8995458 | MW547444 |
| DENV26 | male | Hainan province, Haikou city, Xiuying area | 1 | 06-Sep-19 | SUB8995460 | MW547446 |
| DENV29 | male | Hainan province, Haikou city, Xiuying area | 1 | 08-Sep-19 | SUB8995465 | MW547445 |
| DENV30 | female | Hainan province, Haikou city, Xiuying area | 1 | 08-Sep-19 | SUB8995467 | MW547447 |
| DENV31 | male | Hainan province, Haikou city, Xiuying area | 1 | 07-Sep-19 | SUB8995469 | MW547448 |
| DENV32 | male | Hainan province, Haikou city, Xiuying area | 1 | 10-Sep-19 | SUB8995472 | MW547449 |
| DENV34 | male | Hainan province, Haikou city, Xiuying area | 1 | 04-Sep-19 | SUB8995478 | MW550297 |
| DENV35 | male | Hainan province, Haikou city, Xiuying area | 1 | 04-Sep-19 | SUB8995481 | MW548641 |
| DENV37 | female | Hainan province, Haikou city, Xiuying area | 1 | 09-Sep-19 | SUB8995486 | MW548640 |
| DENV38 | male | Hainan province, Haikou city, Xiuying area | 1 | 09-Sep-19 | SUB8995489 | MW547451 |
| DENV40 | male | Hainan province, Haikou city, Xiuying area | 1 | 09-Sep-19 | SUB8995492 | MW547452 |
| DENV41 | female | Hainan province, Haikou city, Xiuying area | 1 | 09-Sep-19 | SUB8995495 | MW547454 |
| DENV44 | female | Hainan province, Haikou city, Xiuying area | 1 | 07-Sep-19 | SUB8995500 | MW547455 |
| DENV46 | male | Hainan province, Haikou city, Xiuying area | 1 | 07-Sep-19 | SUB8995503 | MW548653 |
| DENV47 | male | Hainan province, Haikou city, Meilan area | 1 | 10-Sep-19 | SUB8995508 | MW548652 |
| DENV50 | male | Hainan province, Haikou city, Xiuying area | 1 | 09-Sep-19 | SUB8995516 | MW547457 |
| DENV52 | female | Hainan province, Haikou city, Xiuying area | 1 | 09-Sep-19 | SUB8995538 | MW547461 |
| DENV53 | male | Hainan province, Haikou city, Xiuying area | 1 | 11-Sep-19 | SUB8995539 | MW547462 |
| DENV54 | female | Hainan province, Haikou city, Xiuying area | 1 | 12-Sep-19 | SUB8995543 | MW547463 |
| DENV55 | female | Hainan province, Haikou city, Meilan area | 1 | 09-Sep-19 | SUB8995544 | MW547465 |
| DENV56 | male | Hainan province, Haikou city, Xiuying area | 1 | 09-Sep-19 | SUB8995545 | MW547464 |
| DENV57 | male | Hainan province, Haikou city, Xiuying area | 1 | 10-Sep-19 | SUB8995547 | MW547466 |
| DENV58 | male | Hainan province, Haikou city, Xiuying area | 1 | 10-Sep-19 | SUB8995548 | MW547467 |
| DENV59 | male | Hainan province, Haikou city, Xiuying area | 1 | 10-Sep-19 | SUB8995549 | MW547468 |
| DENV62 | male | Hainan province, Haikou city, Xiuying area | 1 | 13-Sep-19 | SUB8995552 | MW547470 |
| DENV63 | male | Hainan province, Haikou city, Xiuying area | 1 | 15-Sep-19 | SUB8995555 | MW547471 |
| DENV64 | female | Hainan province, Haikou city, Xiuying area | 1 | 15-Sep-19 | SUB8995556 | MW547473 |
| DENV65 | female | Hainan province, Haikou city, Xiuying area | 1 | 16-Sep-19 | SUB8995557 | MW547472 |
| DENV66 | male | Hainan province, Haikou city, Qiongshan area | 1 | 12-Sep-19 | SUB8995558 | MW547475 |
| DENV68 | male | Hainan province, Haikou city, Xiuying area | 1 | 15-Sep-19 | SUB8995560 | MW547474 |
| DENV70 | female | Hainan province, Haikou city, Xiuying area | 1 | 14-Sep-19 | SUB8995561 | MW547484 |
| DENV81 | male | Hainan province, Haikou city, Xiuying area | 1 | 10-Sep-19 | SUB8995570 | MW547483 |
| DENV82 | male | Hainan province, Haikou city, Xiuying area | 1 | 11-Sep-19 | SUB8995572 | MW547482 |
| DENV84 | male | Hainan province, Haikou city, Xiuying area | 1 | 12-Sep-19 | SUB8995574 | MW547485 |
| DENV85 | male | Hainan province, Haikou city, Meilan area | 1 | 12-Sep-19 | SUB8998294 | MW547511 |
| DENV86 | female | Hainan province, Wanning city | 1 | 13-Sep-19 | SUB8998296 | MW547510 |
| DENV87 | male | Hainan province, Haikou city, Xiuying area | 1 | 13-Sep-19 | SUB8998297 | MW547512 |
| DENV91 | male | Hainan province, Haikou city, Xiuying area | 1 | 12-Sep-19 | SUB8998299 | MW547513 |
| DENV92 | female | Hainan province, Haikou city, Xiuying area | 1 | 11-Sep-19 | SUB8998301 | MW547516 |
| DENV94 | male | Hainan province, Haikou city, Xiuying area | 1 | 14-Sep-19 | SUB8998305 | MW547515 |
| DENV99 | male | Hainan province, Haikou city, Xiuying area | 1 | 13-Sep-19 | SUB8998309 | MW547517 |
| DENV100 | male | Hainan province, Haikou city, Xiuying area | 1 | 14-Sep-19 | SUB8998311 | MW547518 |
| DENV102 | male | Hainan province, Wenchang city | 1 | 13-Sep-19 | SUB8998313 | MW547520 |
| DENV108 | male | Hainan province, Haikou city, Xiuying area | 1 | 13-Sep-19 | SUB8998317 | MW547521 |
| DENV110 | female | Hainan province, Haikou city, Xiuying area | 1 | 13-Sep-19 | SUB8998319 | MW547523 |
| DENV111 | male | Hainan province, Haikou city, Xiuying area | 1 | 14-Sep-19 | SUB8998321 | MW547524 |
| DENV114 | male | Hainan province, Haikou city, Xiuying area | 1 | 19-Sep-19 | SUB8998322 | MW547528 |
| DENV115 | male | Hainan province, Haikou city, Xiuying area | 1 | 19-Sep-19 | SUB8998329 | MW547530 |
| DENV116 | male | Hainan province, Haikou city, Xiuying area | 1 | 19-Sep-19 | SUB8998333 | MW547531 |
| DENV117 | male | Hainan province, Haikou city, Xiuying area | 1 | 17-Sep-19 | SUB8998334 | MW547532 |
| DENV119 | female | Hainan province, Haikou city, Xiuying area | 1 | 18-Sep-19 | SUB8998337 | MW547533 |
| DENV120 | male | Hainan province, Haikou city, Xiuying area | 1 | 10-Sep-19 | SUB8999322 | MW549052 |
| DENV122 | male | Hainan province, Haikou city, Xiuying area | 1 | 21-Sep-19 | SUB8998339 | MW549200 |
| DENV123 | male | Hainan province, Haikou city, Xiuying area | 1 | 20-Sep-19 | SUB8998341 | MW549622 |
| DENV125 | male | Hainan province, Haikou city, Xiuying area | 1 | 21-Sep-19 | SUB8998345 | MW549208 |
| DENV126 | female | Hainan province, Haikou city, Xiuying area | 1 | 21-Sep-19 | SUB8998348 | MW547534 |
| DENV127 | male | Hainan province, Haikou city, Xiuying area | 1 | 20-Sep-19 | SUB8998352 | MW549323 |
| DENV128 | male | Hainan province, Danzhou city | 1 | 20-Sep-19 | SUB8998353 | MW549042 |
| DENV129 | male | Hainan province, Haikou city, Xiuying area | 1 | 19-Sep-19 | SUB8998354 | MW547535 |
| DENV130 | female | Hainan province, Haikou city, Xiuying area | 1 | 19-Sep-19 | SUB8998357 | MW549783 |
| DENV131 | male | Hainan province, Haikou city, Xiuying area | 1 | 20-Sep-19 | SUB8998359 | MW549871 |
| DENV132 | female | Hainan province, Haikou city, Xiuying area | 1 | 19-Sep-19 | SUB8998362 | MW549782 |
| DENV138 | male | Hainan province, Haikou city, Xiuying area | 1 | 20-Sep-19 | SUB8998368 | MW549872 |
| DENV143 | male | Hainan province, Haikou city, Xiuying area | 1 | 23-Sep-19 | SUB8998371 | MW547565 |
| DENV148 | female | Hainan province, Haikou city, Xiuying area | 1 | 25-Sep-19 | SUB8998369 | MW547769 |
| DENV150 | female | Hainan province, Haikou city, Xiuying area | 1 | 24-Sep-19 | SUB8998370 | MW547770 |
| DENV151 | female | Hainan province, Haikou city, Xiuying area | 1 | 21-Sep-19 | SUB8998391 | MW553038 |
| DENV172 | male | Hainan province, Haikou city, Xiuying area | 1 | 15-Oct-19 | SUB8998392 | MW547771 |
| DENV176 | female | Hainan province, Wanning city | 1 | 16-Oct-19 | SUB8998394 | MW547774 |
| DENV180 | male | Hainan province, Lingshui city | 1 | 15-Oct-19 | SUB8998398 | MW547777 |
| DENV181 | male | Hainan province,Lingshui city | 1 | 14-Oct-19 | SUB8998399 | MW547776 |
| DENV183 | male | Hainan province,Lingshui city | 1 | 15-Oct-19 | SUB8998400 | MW553078 |
| DENV184 | male | Hainan province,Danzhou city | 1 | 15-Oct-19 | SUB8998404 | MW547778 |
| **Information of the whole genome sequence** | | | | | | |
| Number | Gender | Address | DENV type | Collect time | Submissions | GenBank |
| DENV3 | female | Hainan province, Haikou city, Xiuying area | 1 | 05-Sep-19 | SUB8998405 | MW553079 |
| DENV5 | female | Hainan province, Haikou city, Xiuying area | 1 | 07-Sep-19 | SUB8998409 | MW547779 |
| DENV33 | male | Hainan province, Haikou city, Xiuying area | 1 | 10-Sep-19 | SUB8998411 | MW547780 |
| DENV42 | female | Hainan province, Haikou city, Xiuying area | 1 | 11-Sep-19 | SUB8998414 | MW553080 |
| DENV49 | female | Hainan province, Haikou city, Meilan area | 1 | 11-Sep-19 | SUB8998416 | MW547783 |
| DENV121 | female | Hainan province, Chengmai city | 1 | 12-Sep-19 | SUB8998420 | MW548420 |
