# Supplementary material for: The Origin and Molecular Epidemiology of Dengue Fever in Hainan Province, China, 2019
Source: Front Microbiol. 2021 Mar 24;12:657966. doi: 10.3389/fmicb.2021.657966 (PMC8025777; doi:10.3389/fmicb.2021.657966)
Supplement: Supplementary Table 2 — The E gene primers and probes. [file Table_2.docx]

**S2 Table The primers used for Envelope (E) gene to amplify and sequence**

|  | primer | sequence | products length | Notes |
| --- | --- | --- | --- | --- |
| Fragment 1 | D1EF1: | GATTTGGGAGAGTTATGTGAGG | 1480bp | The first amplification |
|  | D1ER1: | TGTTGACTGGTTTTTCTTTGTC |  |  |
|  | D1E1F: | ATAGGAACATCCATCACCCAG | 958bp | The second amplification |
|  | D1E1R: | TCCCTTTTAGAGTCAGTTTGTCC |  |  |
| Fragment 2 | D1EF2: | GCAACCATAACACCTCAAGC | 1288bp | The first amplification |
|  | D1ER2: | TCCCAGCAACATCTCCTACA |  |  |
|  | D1E2F: | GCGACRGAAATCCAAACG | 827bp | The second amplification |
|  | D1E2R: | CCRATGGCTGCTGATAGT |  |  |
